# Glucagon‐like peptide‐1 acutely affects renal blood flow and urinary flow rate in spontaneously hypertensive rats despite significantly reduced renal expression of GLP‐1 receptors

**DOI:** 10.14814/phy2.13503

**Published:** 2017-12-12

**Authors:** Jonas Ronn, Elisa P. Jensen, Nicolai J. Wewer Albrechtsen, Jens Juul Holst, Charlotte M. Sorensen

**Affiliations:** ^1^ Department of Biomedical Sciences Faculty of Health Sciences University of Copenhagen Copenhagen Denmark; ^2^ NNF Center for Basic Metabolic Research Faculty of Health Sciences University of Copenhagen Copenhagen Denmark

**Keywords:** Glucagon‐like peptide‐1, glucagon‐like peptide‐1 receptor, hypertension, kidney, SHR

## Abstract

Glucagon‐like peptide‐1 (GLP‐1) is an incretin hormone increasing postprandial insulin release. GLP‐1 also induces diuresis and natriuresis in humans and rodents. The GLP‐1 receptor is extensively expressed in the renal vascular tree in normotensive rats where acute GLP‐1 treatment leads to increased mean arterial pressure (MAP) and increased renal blood flow (RBF). In hypertensive animal models, GLP‐1 has been reported both to increase and decrease MAP. The aim of this study was to examine expression of renal GLP‐1 receptors in spontaneously hypertensive rats (SHR) and to assess the effect of acute intrarenal infusion of GLP‐1. We hypothesized that GLP‐1 would increase diuresis and natriuresis and reduce MAP in SHR. Immunohistochemical staining and in situ hybridization for the GLP‐1 receptor were used to localize GLP‐1 receptors in the kidney. Sevoflurane‐anesthetized normotensive Sprague–Dawley rats and SHR received a 20 min intrarenal infusion of GLP‐1 and changes in MAP, RBF, heart rate, dieresis, and natriuresis were measured. The vasodilatory effect of GLP‐1 was assessed in isolated interlobar arteries from normo‐ and hypertensive rats. We found no expression of GLP‐1 receptors in the kidney from SHR. However, acute intrarenal infusion of GLP‐1 increased MAP, RBF, dieresis, and natriuresis without affecting heart rate in both rat strains. These results suggest that the acute renal effects of GLP‐1 in SHR are caused either by extrarenal GLP‐1 receptors activating other mechanisms (e.g., insulin) to induce the renal changes observed or possibly by an alternative renal GLP‐1 receptor.

## Introduction

Type 2 diabetes mellitus (T2DM) is a metabolic disease which has been declared a global epidemic and the number of patients is increasing. T2DM is often associated with hypertension and both are leading causes in the development of renal failure (Emdin et al. 2015).

Glucagon‐like peptide‐1 (GLP‐1) is an incretin hormone secreted from enteroendocrine L‐cells in the small intestine. GLP‐1 enhances the postprandial insulin release. GLP‐1 is secreted as the active intact GLP‐1 (7–36) amide, but is rapidly degraded to GLP‐1 (9–36) amide by the ubiquitously expressed enzyme dipeptidyl peptidase‐4 (DPP‐4). This results in a very short in vivo half‐life of active GLP‐1 (Deacon et al. 1995). Besides affecting the pancreas, GLP‐1 has a range of extrapancreatic effects including effects in the kidneys and the cardiovascular system (Nauck et al. 2017). It has been demonstrated that GLP‐1 affects blood pressure and renal blood flow (RBF) along with increases in diuresis and natriuresis (Moreno et al. 2002; Gutzwiller et al. 2004; Ban et al. 2008; Crajoinas et al. 2011; Jensen et al. 2015). When used chronically, incretin‐based treatment is suggested to decrease blood pressure in both healthy and diabetic humans (Mistry et al. 2008; Okerson et al. 2010; Wang et al. 2013), although some studies showed no significant effect on blood pressure (Imamura et al. 2013; Skov et al. 2013; Asmar et al. 2015). In rodents, the GLP‐1‐mediated renal and cardiovascular effects seem to depend on the animal model used. In salt‐sensitive Dahl rats fed a high salt diet, GLP‐1 reduced blood pressure and albuminuria, and increased Na^+^ excretion (Yu et al. 2003). The GLP‐1 receptor agonist exendin‐4 inhibited the development of hypertension in salt‐sensitive obese db/db mice (Hirata et al. 2009). In spontaneously hypertensive rats (SHR), DPP‐4 inhibition either increased (Jackson et al. 2015) or decreased blood pressure (Liu et al. 2012), whereas native GLP‐1 was shown not to affect blood pressure (Savignano et al. 2017). DPP‐4 inhibition decreased blood pressure in diabetic Zucker rats (Aroor et al. 2013). Thus, in hypertensive animal models the most pronounced effect of GLP‐1 treatment seems to be a blood pressure reduction, whereas the renal effects have not been studied extensively. DPP‐4 inhibitors are widely used in the treatment of diabetes in order to elevate the endogenous GLP‐1 levels. In healthy humans, GLP‐1 does not affect renal hemodynamics (Skov et al. 2013; Asmar et al. 2015), whereas there may be effects in obese subjects (Gutzwiller et al. 2004). In both healthy and obese insulin‐resistant humans, GLP‐1 induces an increase in diuresis and Na^+^ excretion.

How GLP‐1 mediates its renal effects is not fully understood, but several studies demonstrate that the GLP‐1 receptor is expressed in the kidney in both rodents and humans. However, there is no consensus about the exact location as some authors show expression in the proximal tubules and glomeruli (Crajoinas et al. 2011), whereas others show that it is only expressed in the renal vasculature (Fujita et al. 2013; Pyke et al. 2014; Jensen et al. 2015).

In this study, we investigated the expression of the GLP‐1 receptor in renal tissue from SHR along with renal and cardiovascular effects of acute GLP‐1 treatment in SHR. Our hypothesis was that the GLP‐1 receptor is expressed in the renal vasculature and that GLP‐1 would reduce the hypertension in SHR. We predicted that GLP‐1 would induce a renal vasodilation and increase urinary Na^+^ excretion. Furthermore, we speculated that the vascular effects of GLP‐1 receptor activation work through activation of K_ATP_ channels. The different hypotheses were tested by examining the effect of an acute intrarenal infusion of GLP‐1 on RBF, mean arterial blood pressure (MAP), heart rate (HR), urinary flow rate, and Na^+^ and K^+^ excretion in vivo in anesthetized rats. The involvement of K_ATP_ channels in the GLP‐1‐mediated vascular effects was investigated in vivo and in isolated renal vessels by blocking the K_ATP_ channels using glibenclamide.

## Methods

### GLP‐1 receptor localization in the kidney

#### Immunohistochemical staining of the GLP‐1 receptor

Pancreas and kidney were isolated from both SD rats (*n *=* *3) and SHR (*n *=* *4) and were stored in 4% paraformaldehyde for at least 1 week before further processing. The tissues were embedded in paraffin and histological sections of 4 *μ*m were cut with a microtome. Sections were deparaffinated and rehydrated in double distilled water. Sections were treated with 0.1% pronase in PBS at 37°C for 10 min and rinsed in tris‐buffered saline (TBS). Thereafter, sections were treated with dual block (Dako, Glostrup, Denmark) for 5 min, washed in TBS with 0.05% Tween 20 (TBST), blocked with avidin (Life Technologies, MD) for 10 min, washed with TBST, blocked with biotin for 10 min (Dako), washed with TBST, and preincubated with 3.2 mg/mL poly‐L‐lysine, 3% BSA, 7% donkey serum, and 3% skimmed milk (Dako) for 30 min. Sections were incubated overnight with primary biotinylated GLP‐1 receptor antibody (7F382A, Novo Nordisk). Thereafter, sections were washed three times for 10 min each in TBST followed by treatment with Vectastain ABComplexHRP (Vectorlabs, Herlev, Denmark) in TBS for 30 min and washed again for three times for 10 min each. Sections were incubated with TSA indirect (Perkin Elmer, Skovlunde, Denmark) for 7 min, washed three times for 10 min with TBST followed by treatment with Vectastain ABComplexHRP in TBS for 30 min, and washed three times for 10 min with TBST. Sections were developed with DAB (Dako), counterstained with hematoxylin, rinsed in water, dehydrated, and mounted.

#### In situ hybridization for the GLP‐1 receptor

The paraffin‐embedded pancreatic and kidney tissues from SD rats (*n *=* *3) and SHR (*n *=* *4) were cut with a microtome in histological sections of 3.5 *μ*m. RNA molecules for the GLP‐1 receptor were detected with in situ hybridization using the automated Ventana RNAscope Dicovery platform (Advanced Cell Diagnostics, Hayward, CA) following the manufacturer's description.

### Effects of acute GLP‐1 infusion in vivo

All animal experiments were approved by the Danish National Animal Experiments Inspectorate. Rats were housed in groups of 2–3 rats in individually ventilated cages under a light cycle of 12 h with ad libitum access to normal chow and water.

#### Animal preparation

The experiments were performed in male SHR (*n *=* *10) weighing 270–310 g at the age of 12–14 weeks obtained from Harlan Laboratories, Indianapolis, USA and normotensive male SD rats (*n *=* *19) weighing 290–340 g at the age of 12–14 weeks obtained from Taconic, Lille Skensved, Denmark. Anesthesia was induced with 8% sevoflurane delivered in 35% oxygen and 65% nitrogen. Polyethylene catheters (PE‐10) were placed in the right jugular vein for infusions and in the left carotid artery (PE‐50) for continuous measurement of the systemic blood pressure by a Statham P23‐dB pressure transducer (Gould, Oxnard, CA). A tracheostomy was performed and the rat was connected to and ventilated by a small animal ventilator, tidal volume 1.7–2.1 mL depending on body weight at a frequency of 60 breaths/min. The rat was placed on a heating table to maintain body temperature at 37°C. The final sevoflurane concentration needed to maintain sufficient anesthesia was ∼2%. An intravenous (i.v.) bolus injection of the muscle relaxant cisatracurium besilate (Nimbex; GlaxoSmithKline, Brøndby, Denmark) in 0.5 ml 0.9% saline was administered and followed by continuous i.v. infusion of 0.6 mg/mL at 20 *μ*L/min. Additional saline was given continuously at a rate of 20 *μ*L/min throughout the experiment.

The left kidney was exposed after a laparotomy, which was extended to the left flank. The left femoral artery was catheterized (PE‐10), and the catheter was moved through the aorta into the ostium of the renal artery in order to administer test agents directly into the kidney, thereby minimizing systemic effects of these. To keep the renal catheter open saline was infused at a rate of 10 *μ*L/min. The left ureter was catheterized (PE‐10 connected to PE‐50) to ensure free urine flow. An ultrasonic flow probe (Transonic 1PRB) was placed around the left renal artery in order to measure RBF. The kidney was superfused with heated saline (37°C) throughout the experiment. Rats were allowed to equilibrate for at least 30 min after the surgical procedures before initiation of the experiment.

The intrarenal infusion rate was increased from 10 to 144 *μ*L/min when the test agents were administered in order to ensure a rapid distribution. Renal arterial plasma concentrations of the administered agents were the estimated plasma concentrations, unless otherwise stated. Concentrations were calculated assuming a hematocrit of 40% (Probst et al. 2006) and an average renal plasma flow of 3 mL/min. The sequence of the following treatments was randomized between the rats such that BSA was not always first followed by GLP‐1 etc. It was not possible for all rats to receive all the different treatments. However, all rats received GLP‐1 administered alone. During the experiment, urine was collected in 5 min periods. At the end of each treatment, a blood sample was collected. EGTA (10 *μ*L of 300 mmol/L) was added to the blood samples to prevent coagulation. The blood samples were centrifuged at 5500 *g* for 5 min, and the plasma was kept frozen for later electrolyte measurement.

#### GLP‐1‐mediated effects in vivo in anesthetized rats

We first tested the effect of 20 min intrarenal infusion of vehicle (1% BSA dissolved in saline) to ensure that observed changes in RBF and blood pressure was caused by GLP‐1 and not by the infusion itself (SD rats: *n *=* *6, SHR: *n *=* *5). The acute effect of intrarenal infusion of GLP‐1 was investigated (SD rats: *n *=* *19, SHR: *n *=* *10). GLP‐1 at an estimated renal plasma concentration of 1 nmol/L was infused for 20 min. Urine was collected every 5 min and a blood sample was drawn at the end of the GLP‐1 infusion.

#### Effects of exendin 9‐39 in vivo in anesthetized rats

The acute effect of intrarenal infusion of GLP‐1 was also investigated when GLP‐1 receptors were blocked using the GLP‐1 receptor antagonist exendin 9‐39 (SD rats: *n *=* *6, SHR: *n *=* *6). An intrarenal infusion of exendin 9‐39 at an estimated renal plasma concentration of 100 nmol/L was administered for 10 min prior to GLP‐1 infusion. Exendin 9‐39 infusion was continued during the GLP‐1 infusion. Urine was collected for 5 min intervals, and a blood sample was drawn at the end.

#### Effects of inhibition of K_ATP_ channels in vivo in anesthetized rats

The acute effect of intrarenal infusion of GLP‐1 was investigated after inhibition of K_ATP_‐channels using glibenclamide, a K_ATP_‐channel inhibitor (SD rats: *n *=* *8, SHR: *n *=* *6). An intrarenal infusion of glibenclamide at an estimated renal plasma concentration of 10 *μ*mol/L was administered for 5 min prior to GLP‐1 infusion. This concentration of glibenclamide has previously been used without affecting baseline RBF (Sorensen et al. 2011). Glibenclamide was administered continuously during the GLP‐1 infusion. Urine was collected in 5 min intervals, and a blood sample was drawn at the end.

### Isometric myograph recordings

Isoflurane‐anesthetized SD rats (*n *=* *4) and SHR (*n *=* *5) were killed by cervical dislocation. Kidneys were removed and placed in ice‐cold dissection buffer pH 7.4 [(mmol/L): NaCl 135, KCl 5, MgCl 1, Hepes 10, Glucose 5, CaCl_2_ 1, Albumin 5 g/L]. Interlobar arteries with an inner diameter of 200–400 *μ*m and a length of 1–2 mm were isolated in ice‐cold dissection buffer. Two stainless steel wires (Ø 40 *μ*m) were introduced through the arterial lumen and the arteries were mounted in a wire myograph (Model 620M; Danish Myo Technology, Århus, Denmark). The chamber contained a 37°C PSS solution [(mmol/L): NaCl 130, KCl 4.7, KH_2_PO_4_ 1.18, MgSO_4_ 1.17, NaHCO_3_ 14.9, EDTA 0.026, CaCl_2_ 1.6, Glucose 5.5] gassed with 5% CO_2_ and 95% O_2_ to maintain a constant pH at 7.4. Lab Chart (ADInstruments, Oxford, UK) was used to record isometric tension.

After 30 min the arteries were stretched to L_100_, which is the stretch that generates a force in the vessel wall corresponding to a transmural pressure of 100 mmHg (Mulvany and Halpern 1977). All experiments were performed at 90% of L_100_. The viability was tested by addition of noradrenaline (NE; 10 *μ*mol/L) in a 60 mmol/L K^+^ solution pH 7.4 (KPSS; mmol/L): NaCl 74.7, KCl 60, KH_2_PO_4_ 1.18, MgSO_4_ 1.17, NaHCO_3_ 14.9, EDTA 0.026, CaCl_2_ 1.6, and Glucose 5.5).

After normalization and test of viability, the arteries were allowed to equilibrate for 20 min. Hereafter, the arteries were contracted with NE (1 *μ*mol/L). When a stable constriction was observed, GLP‐1 was added cumulatively to the chamber (1 pM to 1 *μ*mol/L) every 90 seconds. Then, the chambers were washed with PSS. Hereafter, the arteries were incubated with the K_ATP_ channel inhibitor glibenclamide (30 *μ*mol/L for SHR and 0.1 or 10 *μ*mol/L for SD rats) for 30 min. NE (1 *μ*mol/L) was added and the GLP‐1 dose–response curve repeated. The chambers were washed and the arteries equilibrated for 20 min. Hereafter, arteries from SHR were contracted with NE (1 *μ*mol/L) for the same time period as the GLP‐1 administration in order to serve as time control. Due to the lack of effect of GLP‐1, we tested the dilatory responses of renal interlobar arteries from SHR by performing a dose–response curve with ACh (1 nmol/L–1 *μ*mol/L) after preconstriction with 1 *μ*mol/L NE.

### Biochemical analysis

Plasma insulin concentrations were measured using a modified commercial sandwich ELISA (cat. No. 10‐1250‐01, Mercodia, Sweden) targeting “rat insulin” with plasma recoveries of 90 ± 9% as described previously (Wewer Albrechtsen et al. 2016).

### Analysis

For statistical analysis, SigmaPlot software (SyStat Software, Chicago, IL) was used. RBF and mean arterial pressure (MAP) are presented as a mean of the last 30 sec in each time period. Renal vascular resistance (RVR) was calculated as follows: RVR = MAP/RBF.

Urine flow was measured gravimetrically. Concentrations of Na^+^ and K^+^ in urine and plasma were measured by flame photometry (IL243 LED flame photometer, Instrumentation Laboratory, Alleroed, Denmark).

Changes in MAP, RBF, HR, RVR, diuresis, Na^+^ excretion, and K^+^ excretion within the groups were analyzed using one‐way ANOVA for repeated measurements followed by Student–Newman–Keuls (SNK) post hoc test. Differences between the different groups were analyzed using one‐way ANOVA followed by SNK post hoc test. Differences between SD and SHR within same treatment group were analyzed using two‐way ANOVA followed by SNK post hoc test. All results are given as mean ± SEM. *P *= 0.05 were considered significant.

## Results

### GLP‐1 receptor localization in the kidney

#### Immunohistochemistry

**Figure 1:**
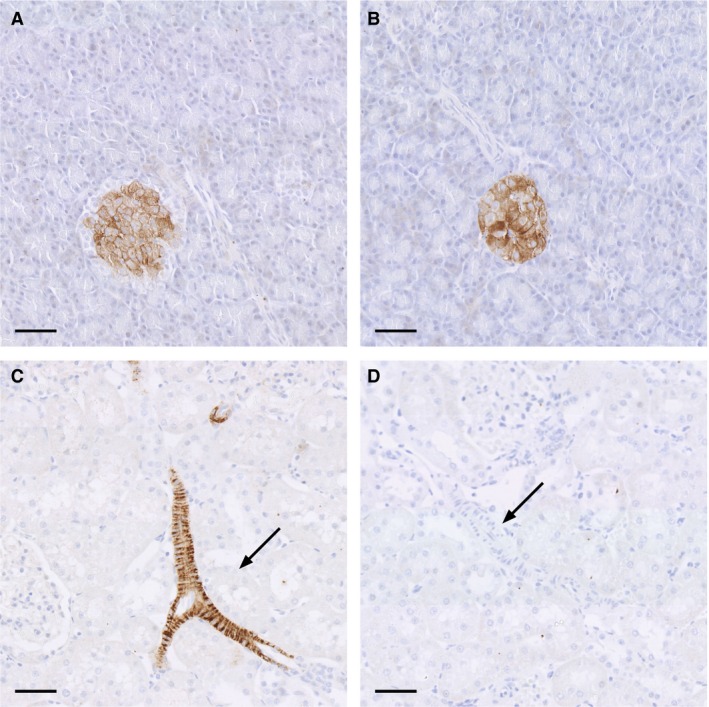
Microphotographs of histological sections, hematoxylin stained, stained for GLP‐1 receptor immunoreactivity in SD pancreas (A), SHR pancreas (B), SD kidney (C), and SHR kidney (D). GLP‐1 receptor immunoreactive cells were localized in the islets of Langerhans in both SD rats (C) and SHR (D). In histological kidney sections from SD (C) there were GLP‐1 receptor immunoreactive cells in the renal vasculature including larger vessels (indicated with an arrow). In histological kidney sections from SHR (D) there were no GLP‐1 receptor immunoreactive cells in the renal vasculature (arrow). Scale bar indicates 50 *μ*mol/L.

In the islets of Langerhans in both SHR rats and SD there was GLP‐1 receptor‐positive immunohistochemical staining (Fig. 1A and B). In the renal tissue from SD rats, there were GLP‐1 receptor immunoreactive cells in the vasculature including the afferent arterioles and larger vessels (Fig. 1C). In SHR, there were no GLP‐1 receptor immunoreactive cells in the renal tissue (Fig. 1D), showing that there are no renal GLP‐1 receptors expressed in SHR.

#### In situ hybridization for the GLP‐1 receptor

**Figure 2:**
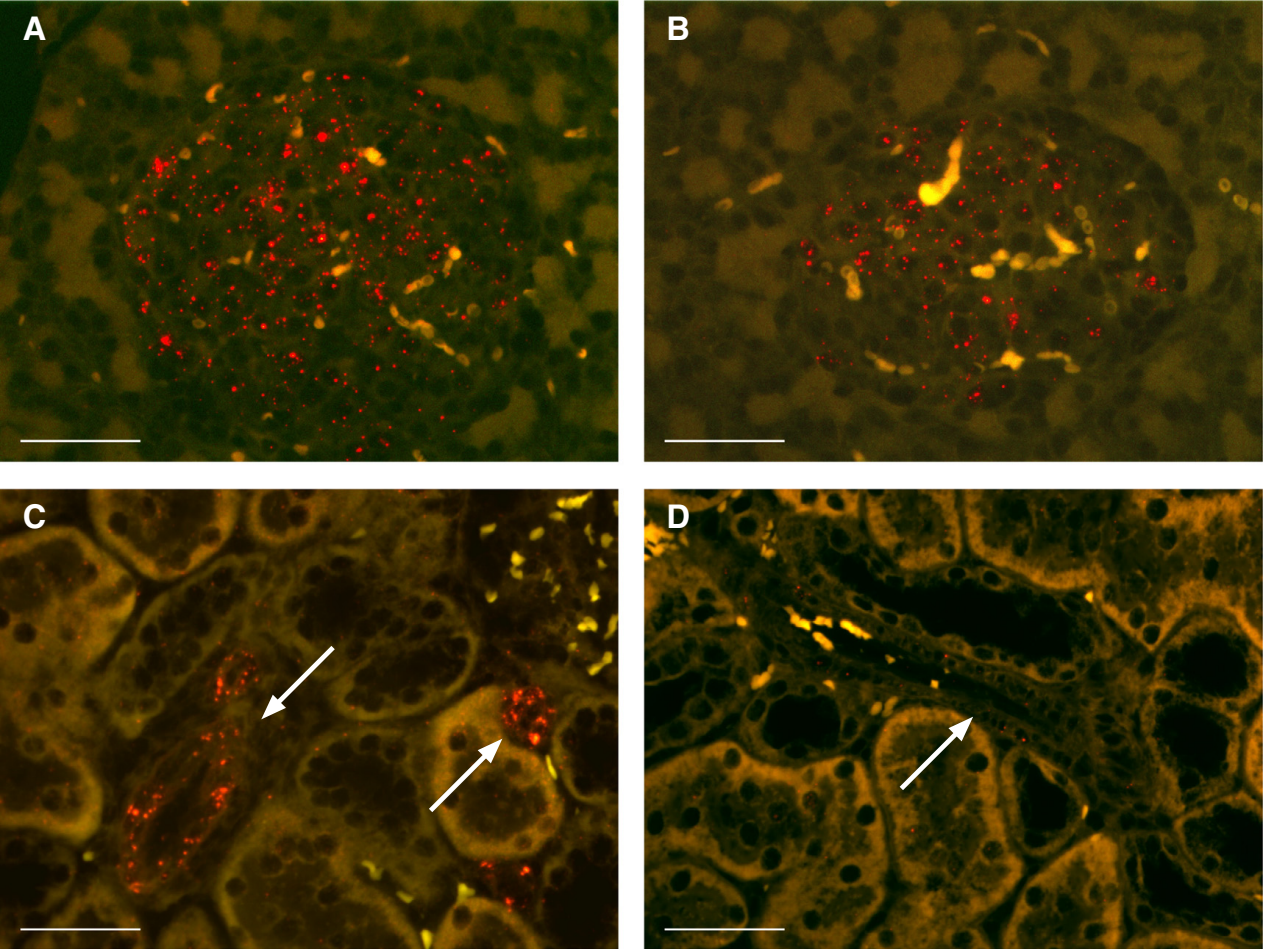
Microphotographs of histological sections, in situ hybridized for the GLP‐1 receptor in SD rat pancreas (red dots) (A), SHR pancreas (B), SD rat kidney (C), and SHR kidney (D). GLP‐1 receptor‐positive staining was located in the islets of Langerhans in both SD rats and SHR (A and B) and in the renal vasculature (indicated with an arrow) in SD rats (C) but not in the renal vasculature (indicated with an arrow) SHR rats (D). Scale bar indicates 50 *μ*mol/L.

There was GLP‐1 receptor positive staining in the islets of Langerhans in both SD rats and SHR (Fig. 2A and B). There was GLP‐1 receptor‐positive staining in the renal vasculature from SD rats (Fig. 2C), whereas there was very low or no GLP‐1 receptor‐positive staining in the vasculature from SHR (Fig. 2D).

### Effects of acute GLP‐1 infusion in vivo

#### Baseline effects of BSA

The physiological parameters from SD and SHR rats receiving BSA, GLP‐1, and exendin 9‐39 + GLP‐1 are shown in Tables 1 and 2 in actual values.

**Table 1:** Physiological status of Sprague–Dawley rats used for 20 min intrarenal administration of GLP‐1

	Control (*n *=* *9)	BSA 20 min	Control (*n *=* *19)	GLP‐1 20 min	Control (*n *=* *6)	GLP‐1 + Ex 20 min
MAP (mmHg)	104 ± 2	103 ± 3	111 ± 2	113 ± 2^b^	117 ± 6	116 ± 6
RBF (mL/min)	7.2 ± 0.7	7.8 ± 0.9	8.6 ± 0.7	9.9 ± 0.8^b^	9.0 ± 1.6	9.3 ± 1.6
HR (BPM)			354 ± 8	356 ± 9	346 ± 10	343 ± 13
*V*_U_ (*μ*L/min)	12.1 ± 1.7	19.1 ± 2.8	30.6 ± 1.9	76.5 ± 10.8^b^	27.6 ± 2.4	53.2 ± 18.1^b^
U_Na+_ (*μ*mol/min)	2.8 ± 1.3	5.1 ± 1.3	4.0 ± 1.2	12.6 ± 1.8^b^	3.6 ± 1.0	8.2 ± 4.4^a^
U_K+_(*μ*mol/min)	2.7 ± 0.2	2.3 ± 0.2	3.5 ± 0.3	3.6 ± 0.9	2.7 ± 0.3	2.5 ± 0.2
Insulin (*μ*g/L)		2.1 ± 0.2		2.7 ± 0.4		

Values are mean ± SE. HR, heart rate; BPM, beats per min; *V*_u_: urine flow rate; U_Na+_, urinary sodium excretion rate; U_K+_, urinary potassium excretion rate.

a *P *<* *0.05 versus control.

b *P *<* *0.01 versus control.

**Table 2:** Physiological status of SHR rats used for 20 min intrarenal administration of GLP‐1

	Control (*n *=* *5)	BSA 20 min	Control (*n *=* *10)	GLP‐1 20 min	Control (*n *=* *6)	GLP‐1 + Ex 20 min
MAP (mmHg)	158 ± 9	159 ± 8	167 ± 9	178 ± 10^a^	167 ± 5	167 ± 5
RBF (mL/min)	4.1 ± 0.4	3.9 ± 0.5	4.4 ± 0.7	5.1 ± 0.8^a^	4.4 ± 0.4	4.6 ± 0.5
HR (BPM)			288 ± 6	287 ± 6	286 ± 7	288 ± 6
*V*_U_ (*μ*L/min)	10.1 ± 2.3	13.3 ± 3.2	13.9 ± 1.9	24.8 ± 7.8^a^	13.0 ± 3.0	19.5 ± 3.1^a^
U_Na+_ (*μ*mol/min)	1.8 ± 0.6	2.5 ± 0.8	2.0 ± 0.5	4.2 ± 1.3^a^	2.6 ± 0.9	3.7 ± 0.9^a^
U_K+_(*μ*mol/min)	1.2 ± 0.2	1.1 ± 0.2	1.3 ± 0.1	1.3 ± 0.1	1.3 ± 0.2	1.4 ± 0.2
Insulin (*μ*g/L)		1.8 ± 0.2		4.2 ± 0.8^b^		

Values are mean ± SE.

HR, heart rate; BPM, beats per min; *V*_u_, urine flow rate; U_Na+_, urinary sodium excretion rate; U_K+_, urinary potassium excretion rate.

a *P *<* *0.01 versus control.

b *P *<* *0.05 versus BSA.

**Figure 3:**
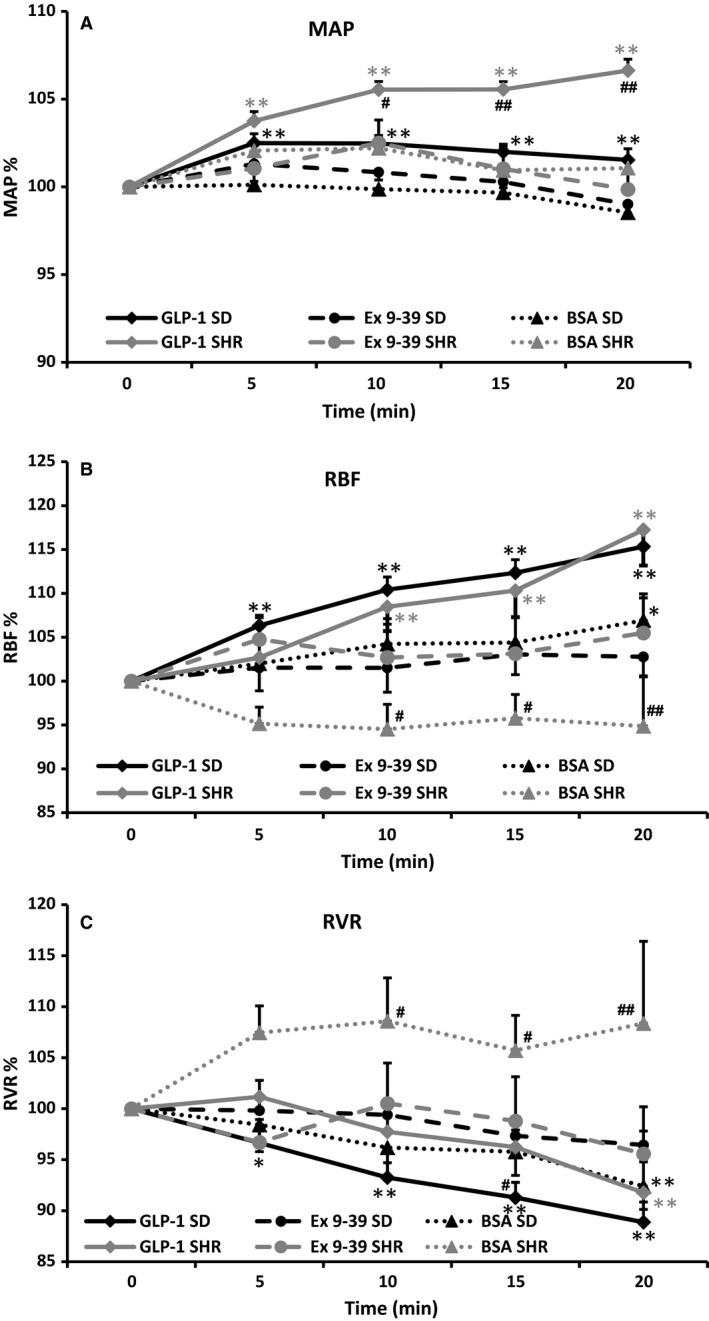
Changes in mean arterial pressure (MAP; A), renal blood flow (RBF; B), and renal vascular resistance (RVR; C) in SD and SHR rats after intrarenal infusion of 1% BSA (*n *=* *9 and *n *=* *5), 1 nmol/L GLP‐1 (*n *=* *19 and *n *=* *10), GLP‐1 and exendin 9‐39 (100 nmol/L; *n *=* *6 and *n *=* *6). Values are normalized means ± SE. *P <* *0.05 versus time 0; **P <* *0.01 versus time 0; #*P *<* *0.05 SD versus SHR within same treatment group; ##*P *<* *0.01 SD versus SHR within same treatment group.

BSA administered alone for 20 min did not induce significant changes in MAP in either rat strain (Fig. 3A). BSA did induce a small significant change in RBF and RVR in SD rats after 20 min (Fig. 3B and C) when compared with baseline at time 0 min. In SHR BSA infusion over 20 min reduced RBF and increased RVR but due to variation these changes were not significant when compared with baseline at time 0 min.

#### GLP‐1‐mediated effects on MAP

In both SD and SHR rats, GLP‐1 mediated a small but significant increase in MAP (102 ± 3% and 107 ± 6%, respectively; *P *<* *0.01; Fig. 3A). Compared to the increase in SD the effect on MAP was significantly larger in SHR after 10 min (*P *<* *0.05). After pretreatment with exendin 9‐39, there was no increase in MAP in either strain. During glibenclamide administration, GLP‐1 still increased MAP in both strains (SD: 103 ± 2%; SHR: 106 ± 2%). Pretreatment with glibenclamide in SHR appeared to increase MAP by itself (from 169 ± 15 mm Hg to 180 ± 12 mmHg), elevating the baseline value measured before GLP‐1 administration.

#### GLP‐1‐mediated effects on RBF

In SD rats, intrarenal infusion of 1 nmol/L GLP‐1 increased RBF to 115 ± 12% (*P *<* *0.01) in normotensive SD rats (*n *=* *19: Fig. 3B). The GLP‐1‐mediated increase in RBF was significant after 5 min and persisted until the end of the GLP‐1 infusion when compared with baseline at time 0 min. Pretreatment with 100 nmol/L exendin 9‐39 (*n *=* *6) abolished the GLP‐1‐mediated effect on RBF (Fig. 3B).

**Figure 4:**
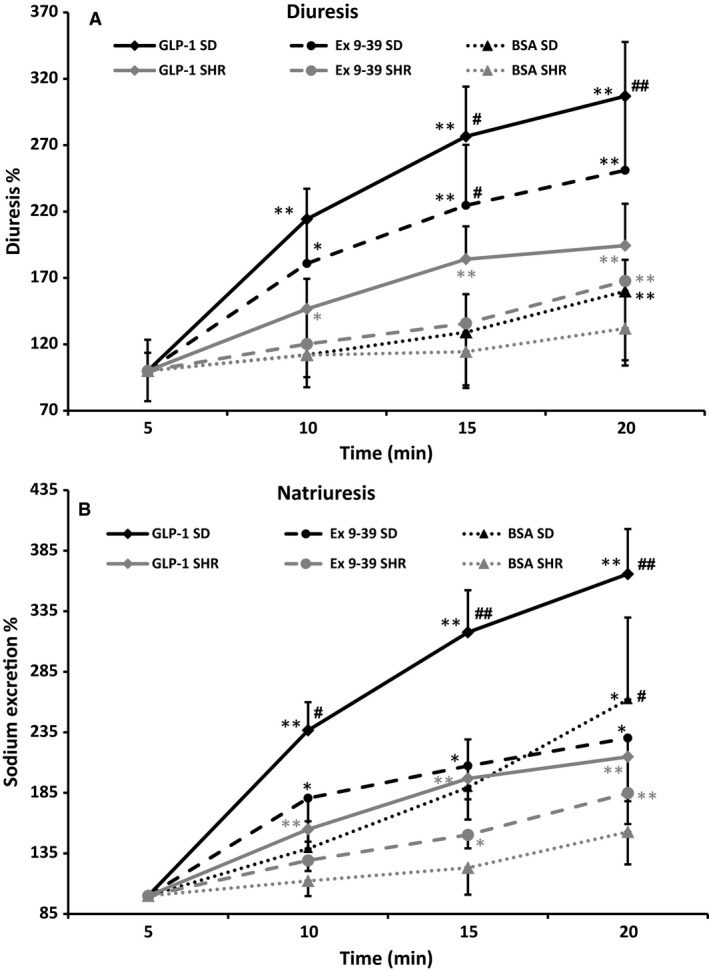
Changes in urine and sodium excretion in SD and SHR rats after intrarenal infusion of 1% BSA (*n *=* *6 and *n *=* *5), 1 nmol/L GLP‐1 (*n *=* *19 and *n *=* *10), GLP‐1 and exendin 9‐39 (100 nmol/L; *n *=* *6 and *n *=* *6). *P <* *0.05 versus time 0; **P <* *0.01 versus time 0; #*P *<* *0.05 SD versus SHR within same treatment group; ##*P *<* *0.01 SD versus SHR within same treatment group.

In SHR, intrarenal infusion of 1 nmol/L GLP‐1 increased RBF to 117 ± 13% (*n *=* *10; Fig. 4; *P *<* *0.01). The increase was significant after 10 min infusion and persisted until the end of the GLP‐1 infusion. Pretreatment with 100 nmol/L exendin 9‐39 abolished the increase in RBF (*n *=* *6; Fig. 3B).

When the K_ATP_ channel inhibitor glibenclamide (10 *μ*mol/L) was administered as pretreatment, GLP‐1 still mediated a significant increase in RBF to 118 ± 15% in SD (*n *=* *8, results not shown) and 115 ± 12% in SHR (*n *=* *6; results not shown), respectively.

#### GLP‐1‐mediated effects on RVR and HR

GLP‐1 mediated a decrease in RVR in both strains (to 89 ± 9% in SD rats (*P *<* *0.01) and to 92 ± 10% in SHR (*P *<* *0.01); Fig. 3C). After pretreatment with exendin 9‐39 no decrease in RVR was seen (Fig. 3C). Coadministration of glibenclamide abolished the decrease in RVR in SHR due to the increasing effect on MAP, but not in the SD rats (results not shown).

GLP‐1, exendin 9‐39, and glibenclamide did not alone or in combination with one another affect HR (Tables 1 and 2).

#### GLP‐1‐mediated effects on diuresis and Na^+^ excretion

BSA administration for 20 min increased diuresis and Na^+^ excretion slightly but significantly in SD rats, but not in SHR (Fig. 4A and B). However, these increases were smaller than the increases observed during administration of GLP‐1.

GLP‐1 mediated a significant increase in diuresis in both SD rats and SHR (307 ± 41% and 194 ± 31% in SD and SHR, respectively, Fig. 4A). However, the diuretic effect of GLP‐1 was significantly larger in SD (*P *<* *0.01). Exendin 9‐39 reduced the GLP‐1‐mediated diuresis (251 ± 55% and 168 ± 22% in SD and SHR, respectively, Fig. 4A). Glibenclamide seemed to augment the GLP‐1‐mediated diuresis in SHR but no SD (data not shown).

GLP‐1 mediated a significant increase in urinary Na^+^ excretion in both strains (366 ± 37% and 215 ± 27%, respectively, Fig. 4B). Again the natriuretic effect was significantly larger in SD rats (*P *<* *0.01). When GLP‐1 receptors were blocked with exendin 9‐39, the natriuretic effect of GLP‐1 was reduced significantly in SD rats (230 ± 52%; *P *<* *0.05), but not in SHR (185 ± 26%). In both strains, the GLP‐1‐mediated increase in urinary Na^+^ excretion was maintained during cotreatment with glibenclamide (data not shown).

There were no GLP‐1‐mediated effects on the urinary K^+^ excretion in any of the groups (Table 1 and 2).

#### GLP‐1‐mediated effects on insulin release

GLP‐1 mediated an increase in plasma insulin after 20 min which was only significant in SHR rats (Tables 1 and 2).

### GLP‐1‐mediated effects on renal arteries ex vivo

##### Isometric myograph recordings

**Figure 5:**
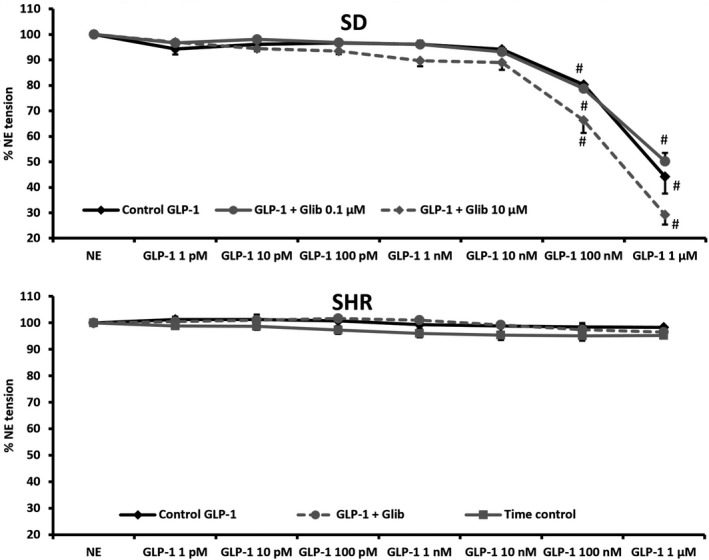
Dose–response curve of increasing concentrations of GLP‐1 in untreated renal interlobar arteries and arteries pretreated with glibenclamide. Arteries were preconstricted with 1 *μ*mol/L NE. Arteries were isolated from (A) SD rats (*n *=* *4) and B) SHR (*n *=* *5). Time control curve for SHR is included for reference. #, *P *<* *0.01 versus NE.

In renal interlobar arteries from SD rats, GLP‐1 (1 pM to 1 *μ*mol/L) had a significant vasodilatory effect at 100 nmol/L and 1 *μ*mol/L (Fig. 5A; *P *<* *0.01). Pretreatment with 0.1 *μ*mol/L or 10 *μ*mol/L glibenclamide did not reduce the vasodilatory effect of GLP‐1 (Fig. 5A). In interlobar arteries from SHR, GLP‐1 did not affect the vascular tone elicited by 1 *μ*mol/L NE (Fig. 5B). The arteries lost approximately 5% of their initial NE‐induced tone over time (Fig. 5B). Glibenclamide (30 *μ*mol/L) had no effect on vascular tone in SHR either (Fig. 5B).

To verify that the renal SHR arteries responded normally, the effect of endothelial activation using ACh (1 nmol/L–1 *μ*mol/L) was also tested. At 1 *μ*mol/L ACh the NE induced constriction was reduced by 49 ± 5% (results not shown).

## Discussion

This study demonstrates that intrarenal infusion of GLP‐1 in normotensive SD rats and hypertensive SHR increases RBF, MAP, diuresis, and Na^+^ excretion. However, the diuretic and natriuretic effect in SHR was attenuated, whereas the effect on MAP and plasma insulin concentrations was slightly increased. The effects of GLP‐1 on RBF and MAP in SD rats and SHR were abolished by exendin 9‐39 showing that these effects are receptor specific. The diuretic and natriuretic effects were only partly reduced by exendin 9‐39 indicating that these effects are at least partly caused by a secondary mechanism. Immunohistochemical staining and in situ hybridization for the GLP‐1 receptor in kidneys from SHR showed no or very low expression of the GLP‐1 receptor. The GLP‐1 receptor was widely expressed in the entire renal vascular tree in SD rats with no expression in the tubular system. These results suggest that the renal effects of GLP‐1 in SHR do not rely on the presence of renal GLP‐1 receptors. Therefore, it seems likely that the GLP‐1‐mediated effects in SHR are mediated via extrarenal GLP‐1 receptors, for example GLP‐1 receptors in organs responsible for regulation of insulin or ANP or possibly by an alternative renal receptor.

We have previously demonstrated GLP‐1 receptors in the renal vasculature of normotensive rats (Jensen et al. 2015). Liu et al. (2015) reported a reduced expression of GLP‐1 receptors in renal arteries from SHR compared to WKY and also in human renal arteries from hypertensive patients based on immunostainings. Quantification based on immunostaining can, however, be extremely difficult. We found no or very low GLP‐1 receptor expression in the kidney from SHR based on immunohistochemical stainings and in situ hybridization. Savignano et al. (2017) also found a significantly reduced expression of GLP‐1 receptors in renal arteries from SHR measured by RT‐PCR . In accordance with this, we observed no GLP‐1‐mediated vasodilatation in isolated interlobar arteries from SHR rats, whereas renal arteries from SD rats showed a GLP‐1‐mediated vasodilatation. Similar results were found by Savignano et al. (2017) and Liu et al. (2015) who also showed that inhibition of protein kinase C (PKC) restored the GLP‐1 induced vasodilation and the expression of the GLP‐1 receptor in renal vessels from SHR. These observations suggest that renal effects of GLP‐1 in SHR are mainly mediated via mechanisms secondary to nonrenal GLP‐1 receptor activation. These secondary mechanisms could be increased levels of insulin or atrial natriuretic peptide (ANP) both shown to affect renal vascular and tubular function (Kim et al. 2013; Artunc et al. 2016). Indeed, we did find a significant increase in plasma insulin concentrations in SHR after 20 min of intrarenal GLP‐1 infusion to substantiate this. Whether the lack of renal GLP‐1 receptors in SHR is a cause or a consequence of hypertension remains to be established.

In this study, there was a GLP‐1 receptor‐dependent increase in MAP in both SD rats and SHR which was significant after 5 min. Jackson et al. (2015) demonstrated that 3 weeks administration of the DPP‐4‐inhibitor sitagliptin increased MAP in SHR but not in normotensive Wistar–Kyoto rats . However, using a higher concentration of sitagliptin Liu et al. (2012) found a reduction in MAP combined with an increase in RBF . It seems that the GLP‐1‐mediated effect on MAP may dependent on the concentration used. In other hypertensive models such as Angiotensin II induced hypertensive rats (Hirata et al. 2009) and in hypertensive Dahl salt‐sensitive rat (Yu et al. 2003), GLP‐1 agonists have been shown to reduce MAP. In this study, the treatment is acute using native GLP‐1. It is possible that a longer treatment period or another hypertensive model would have revealed a reduced MAP. In a human study acute treatment with liraglutide significantly increased diastolic and systolic blood pressure in patients with T2DM (Skov et al. 2016).

In vivo we found a GLP‐1 receptor‐dependent increase in RBF in both SD rats and SHR as the response in both strains was abolished by exendin 9‐39. This is in contrast to the findings of Savignano et al. (2017) who found an increase in RBF after a 30 min i.v. infusion of GLP‐1 in normotensive Wistar rats but not in SHR . However, they only measured RBF at the end of the infusion, whereas we measure continuously. Also, our infusion is directly into the renal artery which could result in a higher renal concentration of GLP‐1. Possibly the same mechanisms are not responsible for the GLP‐1‐mediated vasodilatation in vivo of the renal arteries in normo‐ and hypertensive rats. The GLP‐1‐mediated increase in RBF in SHR could possibly be explained by a GLP‐1‐mediated increase in insulin which we also show here. Even though the SHR has been shown to be insulin resistant, insulin still elicits vasodilation in several vascular beds (Potenza et al. 2005). At high concentration insulin also induces an increase in MAP in SHR (Pitre et al. 1996). Several studies have shown a renal vasodilating effect of insulin (Cohen et al. 1989; Stenvinkel et al. 1995; Qadir and Porter 1996) most likely mediated via release of NO (Hayashi et al. 1997; Molinari et al. 2001). The insulin‐mediated renal vasodilation has been found to be associated with reduced tubular Na^+^ reabsorption. Insulin infusion combined with a slight volume load reduced both proximal and distal reabsorption of Na^+^ and water in healthy humans (Norgaard et al. 1991). In our experimental set‐up, the rats receive 144 *μ*L saline pr. min. Thus, the combined effect of a GLP‐1‐mediated insulin release and the volume load could account for the renal vasodilation and the increased diuresis and natriuresis in vivo despite the lack of GLP‐1 receptor expression in renal tissue in SHR. However, other studies have shown that the renal vasodilatory effect of insulin is reduced in SHR compared to normotensive rats (Santure et al. 2000).

In humans, genetic variations in the GLP‐1 receptor have been shown to affect the efficacy of hypoglycemic agents (de Luis et al. 2015; Han et al. 2016). It is possible that the GLP‐1 receptor expressed in SHR is a variant. However, the variations found in humans are one amino acid substitutions and most likely these variants would respond to the staining methods used in this study. Another possible explanation for the GLP‐1‐mediated renal effects in SHR could be a GLP‐1‐mediated increase in ANP (Kim et al. 2013), which is natriuretic in anesthetized rats (de Bold et al. 1981) and has a renal vasodilating effect (Marin‐Grez et al. 1986). The GLP‐1‐induced increase in ANP has not been demonstrated in humans (Asmar et al. 2015).

As previously shown in normotensive rats, GLP‐1 reduces renal vascular resistance significantly in both SD rats and SHR. Interestingly the increase in RBF is combined with an increase in MAP. How this GLP‐1‐mediated increase in MAP is elicited is still unknown but we found no significant changes in HR to account for this, speaking against an involvement of the sympathetic nervous system. However, GLP‐1 has also been shown to increase the release of vasopressin (Bojanowska and Stempniak 2000) concurrent with increases in blood pressure. Pretreatment with a vasopressin receptor (*V*_1_) antagonist inhibited the increase in blood pressure (Isbil‐Buyukcoskun and Gulec 2004). *V*_1_ is primarily expressed on vascular smooth muscle cells and mediates vasoconstriction (Katusic et al. 1984).

Intrarenal infusion of 1 nmol/L GLP‐1 significantly increased RBF and MAP in both rat strains. This was followed by significant increases in diuresis and urinary Na^+^ excretion. In SHR, the natriuretic and diuretic effect was reduced compared to SD rats and is most likely driven by effects elicited by extrarenal GLP‐1 receptors. As with the changes in RBF, both increases in insulin (Norgaard et al. 1991) and ANP (Kim et al. 2013) could account for this.

Exendin 9‐39 abolished the effects of GLP‐1 on RBF and MAP in both strains, but the diuresis and Na^+^ excretion still increased during coadministration of exendin 9‐39. This suggests that the increase in diuresis and Na^+^ excretion is not driven by the hemodynamic changes induces by GLP‐1 alone. A possible explanation may be GLP‐1‐mediated release of vasopressin. Studies show an increased circulating concentration of vasopressin in rats after GLP‐1 injection (Bojanowska and Stempniak 2000). Vasopressin has been show to increase natriuresis in high concentrations via the *V*_1a_ receptor (Perucca et al. 2008). The combined infusion of a *V*_1a_ receptor agonist and GLP‐1 in rats had a cumulative effect on Na^+^ excretion (Kutina et al. 2016). Furthermore, GLP‐1 was able to increase free water clearance even during blockade of the V_2_ receptor (Kutina et al. 2013).

Differences in activity of the sodium‐hydrogen antiporter 3 (NHE3) in the proximal tubule could explain the reduced effect of GLP‐1 on diuresis and urinary Na^+^ excretion in SHR. NHE3 activity is regulated by phosphorylation by PKA (Cabado et al. 1996). Increased phosphorylation reduces NHE3 activity in proximal tubules in rats (Kocinsky et al. 2005). It has been demonstrated that GLP‐1 increases urinary excretion of cAMP in normotensive rats (Crajoinas et al. 2011). Possibly the increased cAMP production after GLP‐1 treatment leads to reduced NHE3 activity and thereby increased diuresis and urinary Na^+^ excretion. Inhibition of GLP‐1 receptors using exendin 9‐39 reduced NHE3 phosphorylation, increased NHE3 activity and resulted in decreased diuresis and urinary Na^+^ excretion (Farah et al. 2015) as also shown in our experiments. Here, we report a lack of renal GLP‐1 receptor expression in SHR and it may be speculated that the generation of cAMP and thus activation of PKA is significantly reduced in SHR. This in turn would increase NHE3 activity to reduce sodium excretion. However, we were not able to fully inhibit the diuretic and natriuretic effect of GLP‐1 using exendin 9‐39 and thus the diuretic and natriuretic effect of GLP‐1 seems very complex and not fully elucidated.

A previous study has suggested that the vasodilating effect seen after GLP‐1 receptor activation is caused by opening of vascular K_ATP_ channels (Green et al. 2008). To test this, we pretreated rats with the putative K_ATP_ inhibitor glibenclamide in a concentration previously shown not to affect RBF in vivo (Sorensen et al. 2011), but to be effective in vitro (Engbersen et al. 2012). Neither in normotensive nor in hypertensive rats, did glibenclamide reduce the effects of GLP‐1. As glibenclamide also increases insulin release (Efendic et al. 1979) it is possible that the observed effect in these experiments is caused by an additional increase in insulin release. Another possibility is that glibenclamide in high concentrations acts as an opener of K_ATP_ channels. However, voltage‐gated potassium channels (K_V_7) have also been suggested to be activated by GLP‐1 receptor activation (Selley et al. 2014). These channels have been shown to be expressed in renal vessels from rats and to participate in maintaining baseline RBF (Salomonsson et al. 2015).

In summary, we show that kidneys from hypertensive SHR rats lack expression of GLP‐1 receptors. This leads to an absence of renal vasodilation in isolated interlobar arteries from SHR. However, intrarenal infusion of GLP‐1 elicits changes in RBF comparable to the changes observed in normotensive rats. These changes could be mediated by insulin or vasopressin. The GLP‐1‐induced increase in urinary flow rate and sodium excretion is reduced in SHR possibly due to reduced vasopressin release or reduced NHE3 activity.

## Conflict of Interest

None declared.
